# Microstructure and Mechanical Properties of Intercritically Treated Grade 91 Steel

**DOI:** 10.3390/ma13183985

**Published:** 2020-09-10

**Authors:** Yiyu Wang, Wei Zhang, Yong Chae Lim, Yanli Wang, Zhili Feng

**Affiliations:** Materials Science and Technology Division, Oak Ridge National Laboratory, Oak Ridge, TN 37830, USA; zhangw4@ornl.gov (W.Z.); limy@ornl.gov (Y.C.L.); wangy3@ornl.gov (Y.W.)

**Keywords:** Grade 91 steel, creep strength, intercritical treatment, microstructure, mechanical properties

## Abstract

Premature creep failures at the intercritical heat affected zone (ICHAZ) of creep-resistant steel weldments have been frequently reported. However, the creep degradation mechanism of different microstructure constituents in ICHAZ is complicated and needs further clarification. In this work, Grade 91 steel was intercritically heat-treated at a temperature (860 °C) between the critical temperatures A_C1_ and A_C3_, and a correlation between microstructure and mechanical properties of the heat-treated specimen was built. The effects of austenitization and tempering resulting from the intercritical treatment (IT) differentiated the local strain energies between the two microstructure constituents: newly transformed martensite (NTM) and over-tempered martensite (OTM). The formation of NTM grains led to a hardness increase from 247 HV0.5 in the base metal to 332 HV0.5 in the IT specimen. The ultimate tensile strength (UTS) increased from 739 MPa in the base metal to 1054 MPa in the IT specimen. Extensive growth of the OTM grains and rapid recovery of NTM grains took place simultaneously in the IT specimen during a typical tempering at 760 °C. These microstructure degradations led to a lowered hardness of 178 HV0.5, a reduced UTS of 596 MPa, and a poor creep resistance with a minimum creep strain rate of 0.49 %/h at 650 °C in an IT + tempering (ITT) specimen.

## 1. Introduction

Nine to twelve percent chromium creep strength-enhanced ferritic (CSEF) steels are widely used to fabricate high-temperature steam components, for example, headers and mainstream pipes, in thermal power plants [1,2,3]. The structural integrity issues of these welded components exposed to severe service conditions (higher steam temperatures and pressures) in advanced ultra-supercritical power plants are drawing increasing attention from safety and maintenance management perspectives. Among these issues, premature failures—especially Type IV cracking at the heat-affected zone (HAZ) of welded joints—significantly shorten the service lifetimes of those components [4,5,6,7]. It is frequently reported that short-term Type IV cracking has occurred in intercritical HAZ (ICHAZ) exposed to peak temperatures between A_C1_ and A_C3_ [8,9]. In general, the intercritical thermal cycle in the ICHAZ has promoted multiple microstructural evolutions occurring simultaneously, including partial austenitization, precipitate dissolution or coarsening, and matrix tempering [10,11,12], which have led to very complicated microstructures [13]. A typical intercritical microstructure features a mixture of untempered martensite, transformed from austenite formed during heating, and overtempered martensite (OTM) originating from the base metal (BM) [14,15]. This weak microstructure in the ICHAZ, which results in faster creep strength degradation, acts as a metallurgical notch across welds. Intercritical microstructure commonly exists in CSEF steel weldments. Besides welding thermal cycles, post-weld heat treatment (PWHT), which is a standard practice in welding of CSEF steels, can also generate an intercritical microstructure in welds [16]. Based on the composition specifications for 9Cr steels in both the ASME B31 [17] and ASTM A335 standards [18], the critical transformation temperatures A_C1_ and A_C3_ can themselves vary by up to 80 °C [19,20]. An improper PWHT temperature may result in intercritial heat treatment of welds instead of conventional tempering [16]. The composition differences between weld metals from standardized filler wires and BMs from different heats add complexity to the selection of PWHT temperature. Therefore, it is worthwhile to further investigate the evolution and deformation behaviors of this intercritial microstructure.

Many of the works consulted observed that faster matrix grain growth and precipitate coarsening led to a lower dislocation density in the ICHAZ than in other regions during creep testing [21,22,23]. However, some links or interactions between these evolution processes remain unknown: (1) how to quantify the relative fractions of two major microstructure constituents, transformed martensite and over-tempered martensite; (2) the role of partial austenitization and subsequent martensitic transformation in the distribution of remaining precipitates; (3) how the two matrix microstructure constituents evolve during PWHT; and (4) the creep deformation behaviors of the two microstructure constituents. This study attempts to provide answers to these questions by building a correlation between the microstructure evolution and the mechanical properties of the intercritical microstructure in Grade 91 steel. The key features of an intercritical microstructure simulated with a customized heat treatment were characterized. Evolutions of the simulated microstructure after a typical PWHT at 760 °C were also studied for comparison. The room temperature tensile strength and high-temperature creep properties of the intercritically treated (IT) microstructure were tested. Creep deformation and rupture mechanisms in CSEF steel welds also are further discussed.

## 2. Materials and Methods

**Figure 1:**
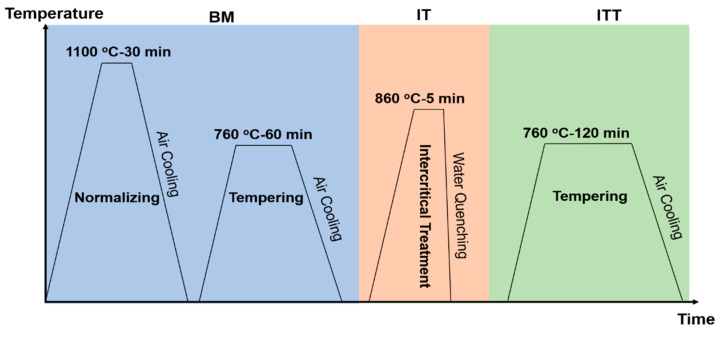
A schematic graph shows the thermal histories of the three specimens (BM, IT, and ITT) before creep testing.

In this study, an 8 mm thick Grade 91 steel plate (Oak Ridge National Laboratory heat No. 30176) was used as the BM and its chemical composition is tabulated in Table 1. The equilibrium phase transformation temperatures A_1_ and A_3_, predicted by Thermo-Calc using the TCFE9 database, were 822 °C and 862 °C, respectively. The thermal history of the three specimens analyzed in this study (BM, IT, and IT plus tempering (ITT)) is shown in Figure 1. The BM was normalized at 1100 °C for 30 min and tempered at 760 °C for 60 min. The IT specimen from the BM was exposed to IT at 860 °C for 5 min. Following the intercritical treatment, additional tempering at 760 °C for 120 min was applied to the ITT specimens.

Mechanical properties, including hardness, room-temperature tensile strength, and high-temperature creep strength, were evaluated for the three specimens. Vickers hardness measurement with a load of 0.5 kgf for 10 s and a spacing of 0.15 mm was also conducted on the three specimens at different stages. An MTS tensile machine was used to evaluate the tensile strength for three conditioned specimens with a crosshead speed of 2 mm/min at room temperature with sub-size specimens (gauge length 50 mm, cross section 4 mm × 6 mm). Creep testing of the three specimens (gauge length 70 mm, cross section 6 mm × 6 mm) was conducted at 650 °C at a stress level of 100 MPa by using an ATS 2330 series lever arm testing system. Hardness mapping was also conducted on the fractured specimens after creep testing.

For microstructural analysis, the BM and heat-treated IT and ITT specimens before and after creep testing were polished using a conventional mechanical polishing method. Grit 320, 600, 800, and 1200 SiC sandpapers were used for grinding. For polishing, a 3 µm and 1 µm diamond suspension, 0.25 µm and 0.05 µm alumina suspension, and 0.02 µm colloidal silica were used. The microstructures of the specimens were characterized with a Zeiss AXIO optical microscope (White Plains, New York, NY, USA), a Hitachi S4800 field-emission scanning electron microscope (FESEM) (Santa Clara, CA, USA), and a JEOL 6500F FESEM (Peabody, MA, USA) equipped with an EDAX electron backscatter diffraction (EBSD) (EDAX, Mahwah, NJ, USA) system. EBSD data acquisition was conducted under 20 kV accelerated voltage and a step size of 0.15 µm. The Analysis OIM (orientation imaging microscopy) software was used to postprocess the EBSD data for orientation maps, grain boundary misorientation maps, and local misorientation maps. The high-angle grain boundary (HAGB, >10°) and low-angle grain boundary (LAGB, 2°–10°) fractions were determined from the misorientation maps. The 10° criterion was chosen based on crystallographic features of low-carbon martensite. A kernel average misorientation (KAM, 0°–5°) map was reconstructed to show the local strain energy distribution. A grain map was used to represent the grain size evolution across different regions.

## 3. Results

### 3.1. Microstructure after Intercritical Heat Treatment

**Figure 2:**
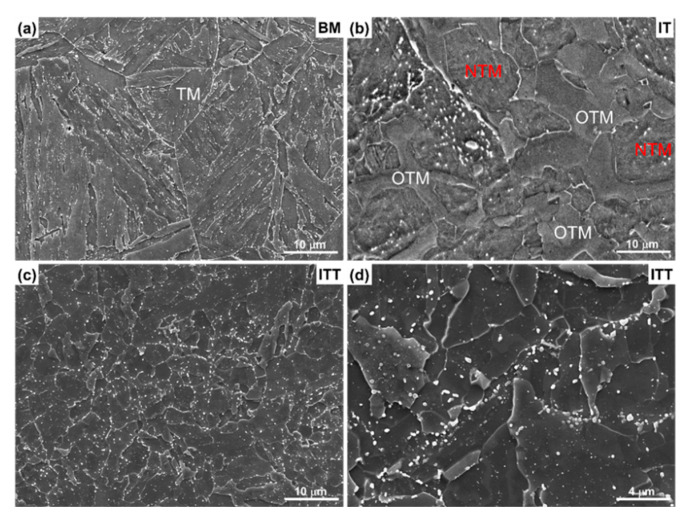
Microstructure of (**a**) BM, (**b**) IT specimens after intercritical heat treatment, and (**c**,**d**) ITT specimen after tempering. Newly transformed martensite (NTM) grains and over-tempered martensite (OTM) grains are highlighted in panel (**b**).

**Figure 3:**
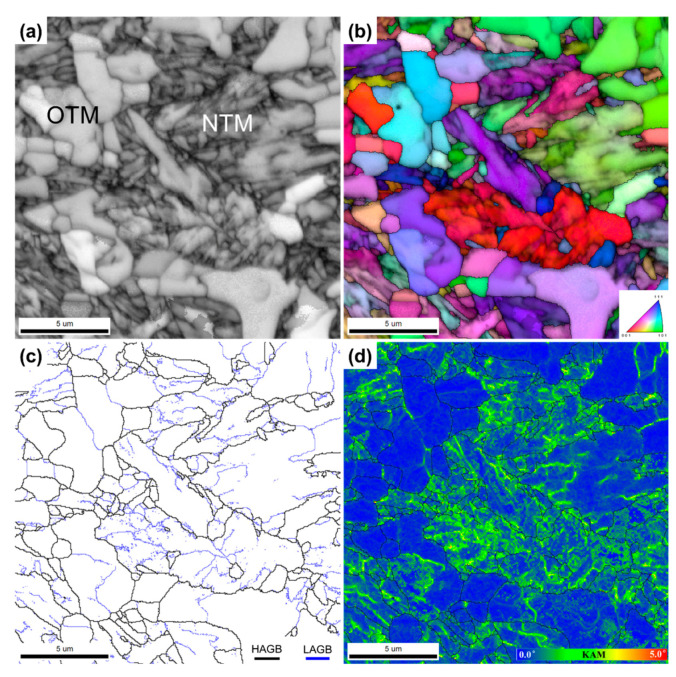
The electron backscatter diffraction (EBSD) analysis shows a mixed microstructure of newly transformed martensite (NTM) and over-tempered martensite (OTM) in the IT specimen after intercritical heat treatment. (**a**) Image quality map; (**b**) inverse pole figure; (**c**) grain boundary map; (**d**) kernel average misorientation map.

Figure 2 presents the microstructural evolution from the BM to the IT and ITT specimens. The BM in Figure 2a shows a typical tempered martensite microstructure with fine precipitates distributed along the prior austenite grain boundaries and martensitic packets/blocks after tempering. These coarse particles are mainly Cr-rich M_23_C_6_ carbides, which have been identified in many previous works [24,25]. After IT, the specimen in Figure 2b exhibits more complex microstructures compared with the BM. It can be observed that partial austenitization and grain growth of tempered martensite took place in the matrix simultaneously. Newly transformed martensite (NTM) grains from the transformed austenite are adjacent to overtempered martensite (OTM) grains. The OTMs are nearly free of internal sub-boundaries and coarse precipitates inside and along the grain boundaries. Dissolution of precipitates led to a much lower precipitate density than in the BM. Undissolved but coarsened precipitates (white dots in Figure 2b) are also observed inside the NTMs. EBSD analysis was conducted to characterize more features of the IT specimen, as shown in Figure 3. The image quality map in Figure 3a shows matrix grains with two morphologies and contrasts. The blocky, bright grains are identified as OTM grains. They exhibit small crystal misorientation with solid inverse pole figure colors (Figure 3b). These OTM grains contain only a limited amount of LAGBs, shown in Figure 3c. The KAM maps in Figure 3b clearly show that the local strain energies inside the OTM grains are extremely low with a KAM value of close to 0°. All these structural features indicate that the OTM grains are no longer martensitic grains but ferritic grains. The fine, dark grains are identified as NTMs. The large misorientation, higher fraction of LAGBs, and high KAM values of these NTMs indicate their high strain energies or dislocation densities.

**Figure 4:**
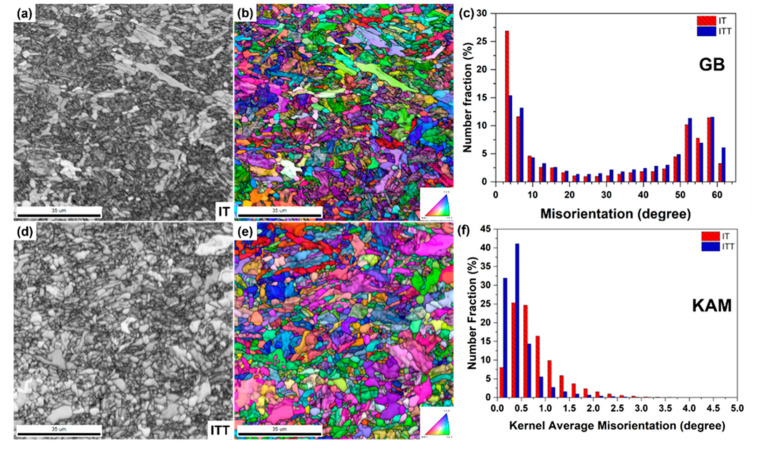
EBSD analysis shows microstructural evolutions in the IT and ITT specimens: (**a**,**d**) image quality maps; (**b**,**e**) inverse pole figures overlapped with image quality maps; (**c**) grain boundary distribution; (**f**) kernel average misorientation (KAM) distribution.

The microstructure of the ITT specimen is shown in Figure 2c,d. Its highly tempered microstructure is quite different from that of the BM and IT specimens. Fine blocky grains instead of lath-like martensite can be observed in the ITT. After tempering, it is not possible to distinguish the NTM and OTM from the IT condition. The number density and size of precipitates has obviously increased, compared with the precipitates observed in the BM and IT. It is also notable that there are two size ranges of precipitates in the ITT. The coarse precipitates are thought to have grown from the undissolved carbides in the IT in Figure 2b. They gather at certain locations, as shown in Figure 2d. Some previous prior austenite grain boundaries from the BM can be traced back by tracking these coarse carbides. Fine precipitates are also observed in the ITT (Figure 2d), which are likely to have formed during tempering. These fine precipitates are distributed nonuniformly inside the matrix grains. The NTM grains likely contain more precipitates than do the OTM grains because they have more boundaries for carbide nucleation. The EBSD analysis illustrated in Figure 4 exhibits more details of microstructural evolution from the IT specimen to the ITT specimen. The measured grain sizes are 1.65 µm for the IT specimen and 1.99 µm for the ITT specimen. The obvious image quality contrast of Figure 4a clearly shows that the IT specimen consists of 68.9% fine NTM grains (dark phase) and 31.1% coarse OTM grains (white phase). After tempering, a fine equiaxed grain is the major microstructure in the ITT specimen shown in Figure 4d. The coarse grains from the OTM are still visible in the matrix. Figure 4c shows grain boundary distributions before and after tempering. The fractions of HAGBs and LAGBs in the IT specimen are 56.9% and 43.1%, respectively. The fractions of the HAGBs and LAGBs in the ITT specimen are 67.1% and 32.9%, respectively. The observed grain recovery and growth in the ITT specimen after tempering contribute to this increased fraction of HAGBs. Meanwhile, peaks of the KAM distribution in the ITT specimen shift to lower KAM values. The normalized KAM values in the IT and ITT specimens are 0.79° and 0.44°, respectively. The lowered KAM value indicates a reduction of the dislocation densities in the ITT specimen owing to the recovery and growth of martensite during tempering.

### 3.2. Mechanical Properties of Heat-Treated Microstructure

**Figure 5:**
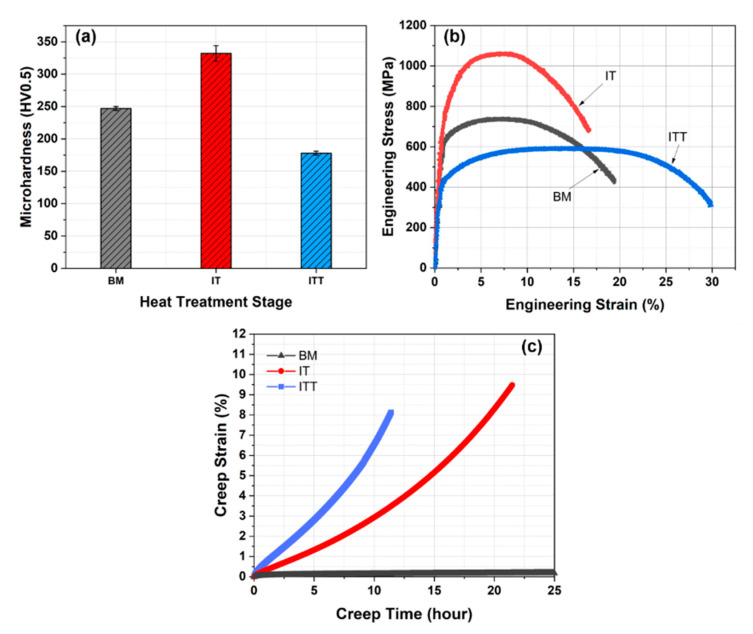
Mechanical properties: (**a**) microhardness, (**b**) tensile strength at room temperature, and (**c**) creep strength tested at 650 °C and a stress of 100 MPa. Note: Only the first part of the entire creep curves were shown in panel (**c**).

The mechanical properties of the BM, IT, and ITT specimens, including hardness, tensile strength at room temperature, and creep strength at elevated temperature (650 °C), were tested and are compared in Figure 5 and Table 2. The BM has a moderate hardness of ~247 HV0.5. The IT exhibits the highest hardness of 332 HV0.5, which is contributed by the hard NTM grains. The additional tempering led to the ITT specimen’s lowest hardness of 178 HV0.5. The typical engineering stress–strain curves of the three specimens are shown in Figure 5b. The yield strength and ultimate tensile strength (UTS) of the BM are 611 MPa and 739 MPa, respectively. The elongation of the BM is 20%. The IT specimen exhibits the greatest strength, with a yield strength of 690 MPa, a UTS of 1054 MPa, and the lowest elongation of 17%. These high strength and low ductility are thought to be contributed by the NTM grains. After additional tempering, the yield strength and UTS of the ITT specimen decrease significantly to 419 MPa and 596 MPa, respectively. Accordingly, the elongation of the ITT specimen increases to 30%.

**Figure 6:**
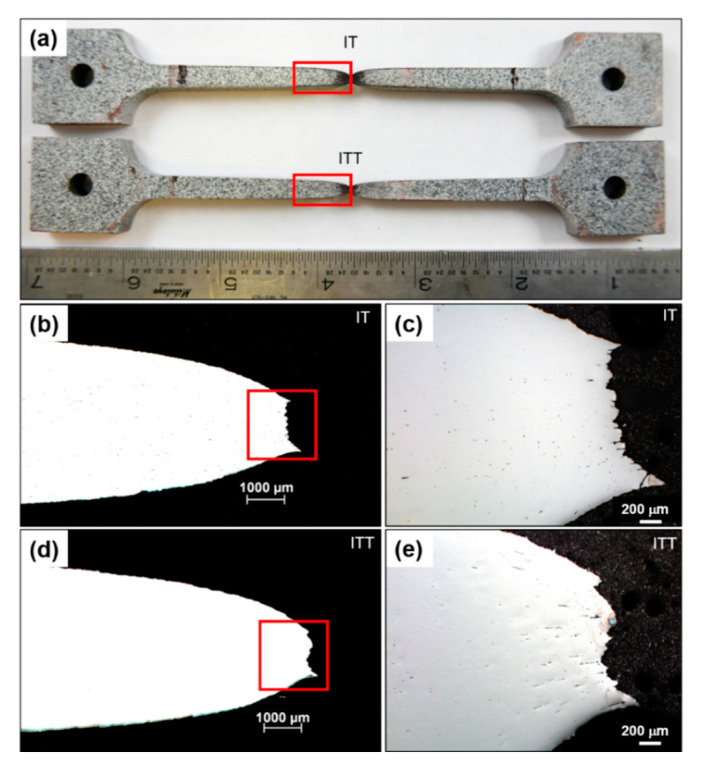
Macrographs and micrographs of the fractured IT and ITT specimens after creep testing. (**a**) Overview of creep fractured IT and ITT specimens; (**b**) cross-section view of fractured IT specimen; (**c**) magnified fracture tip of IT specimen; (**d**) cross-section view of fractured ITT specimen; (**e**) magnified fracture tip of ITT specimen.

**Figure 7:**
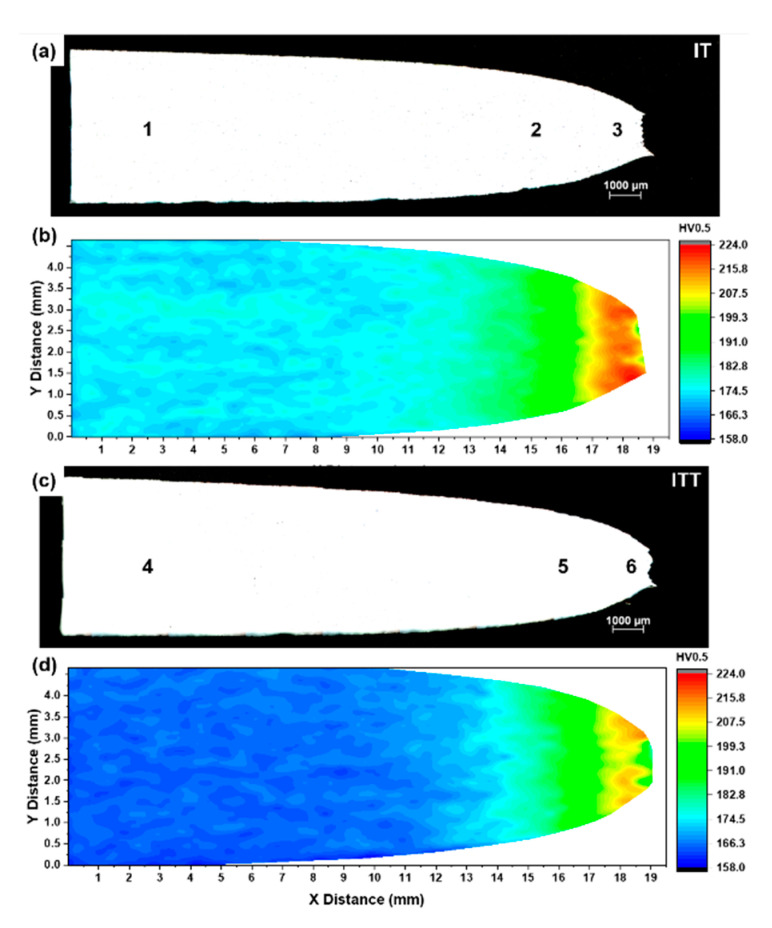
Microhardness distribution across the fractured IT and ITT specimens: (**a**,**c**) optical images and (**b**,**d**) hardness contour maps. The locations 1–6 were marked for microstructure analysis in subsequent section.

The creep strain curves of the three specimens are compared in Figure 5c. The BM shows the highest creep resistance with a minimum creep strain rate of 0.0013 %/h. The creep curves of the IT and ITT specimens are not typical three-stage curves: the specimens quickly go into the tertiary creep stage with a very short secondary creep. The IT and ITT specimens fractured after a total life of 31.8 h and 19.2 h, respectively. The minimum creep strain rate of the ITT specimen is 0.49 %/h, which is twice the 0.24 %/h rate of the IT specimen. The creep-fractured IT and ITT specimens are compared in Figure 6. It is observed that the fracture exhibits ductile features, with obvious necking occurring in the center for both specimens. The cross section views show the fracture surface of the IT specimen is relatively flatter than that of the ITT specimen. Microcracks along the horizontal loading direction are also observed near the fracture surface of the ITT specimen in Figure 6d. To further differentiate the creep fracture behaviors of the IT and ITT specimens, hardness mapping analyses were conducted. Hardness contour maps in Figure 7 show there are three typical hardness regions in each specimen (Regions 1–3 in IT specimen and Region 4–6 in ITT specimen). Region 1 in the IT specimen has a hardness of ~175 HV0.5, which is significantly reduced from the 332 HV0.5 before creep testing. As a result of severe creep deformation, the hardness values in Regions 2 and 3 increase to 191 HV0.5 and 215 HV0.5, respectively. Region 4 in the ITT specimen has the lowest hardness of ~160 HV0.5 after creep testing. Region 5 has comparable hardness to Region 2. Region 6 has a hardness of 205 HV0.5, which is lower than that in Region 3.

### 3.3. Microstructure after Mechanical Testing

**Figure 8:**
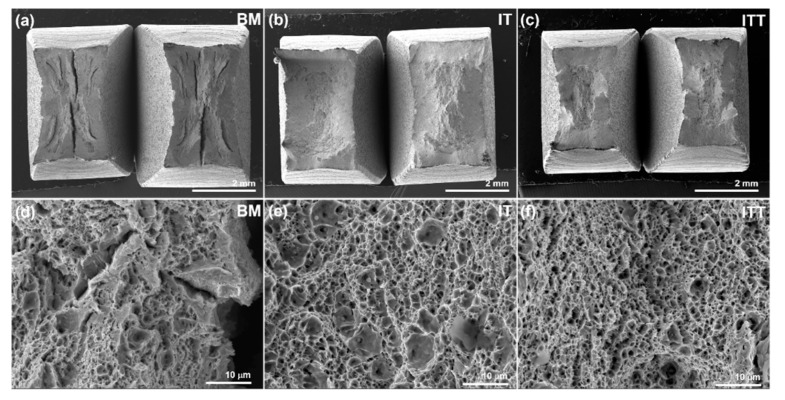
Scanning electron microscope images showing fractography of three specimens after tensile testing. (**a**,**d**) BM; (**b**,**e**) IT; (**c**,**f**) ITT.

Figure 8 compares the fracture surfaces of three specimens after tensile testing. The three specimens are different in appearance in terms of topography and morphology. Figure 8a shows the BM specimen has the roughest surface among the three. Large cracks on both surfaces indicate the tearing fracture mode of the specimen. A high-magnification scanning electron microscope (SEM) image in Figure 8d shows the BM surface is a ductile fracture surface with many dimples. It is notable that microcracks with split tearing features are also observed. The microstructure of the coarse tempered martensite and prior austenite grain boundaries in the BM may have led to the ductile fracture and tearing cracks, respectively. The IT specimen shows the smallest cross section reduction owing to its lowest elongation (17%) and the largest cup/cone depth, consistent with its highest UTS (1054 MPa). The micrograph in Figure 8e also shows a ductile fracture with dimples. Large microvoids with inclusion cores are observed on the fracture surfaces. Figure 8c shows the ITT specimen has the largest cross section reduction, corresponding to the largest elongation (30%). The fine dimples on the fracture surfaces confirm it has the highest ductility among the three.

**Figure 9:**
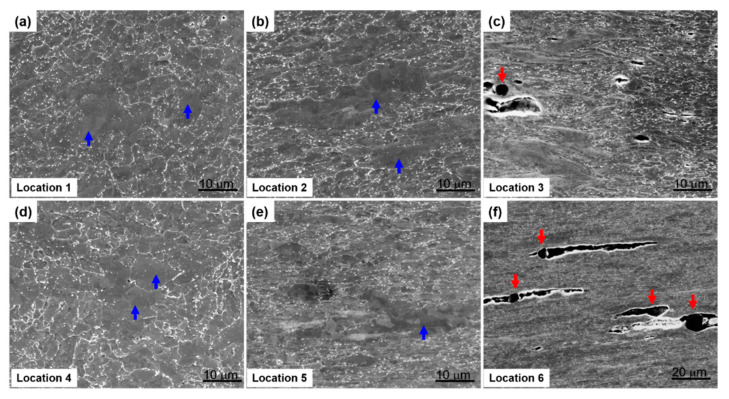
Scanning electron microscope images showing microstructural evolutions in the fractured specimens after creep testing (longitudinal view). The analyzed locations are correlated with the locations marked in Figure 7. (**a**–**c**) IT specimen; (**d**–**f**) ITT specimen. Coarse grains and inclusions are highlighted by blue arrows and red arrows in the figures, respectively.

**Figure 10:**
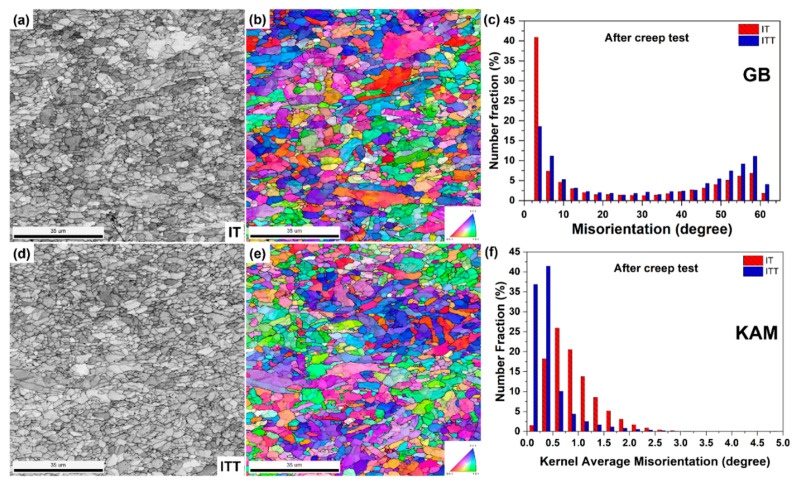
EBSD analyses of the unfractured regions in the IT and ITT specimens after creep testing. (**a**) Image quality map and (**b**) inverse pole figure of location 1 marked in Figure 7; (**c**) grain boundary distribution; (**d**) image quality map and (**e**) inverse pole figure of location 1 marked in Figure 7; (**f**) kernel average misorientation distribution.

The microstructures of the three creep-ruptured specimens are characterized and compared in Figure 9. The analyzed locations (1–6) are correlated with the locations marked in Figure 7. For locations 1 and 4, the matrix consists of both fine equiaxed grains and coarse grains with a low density of precipitates. The EBSD analyses pictured in Figure 10 further compare the microstructures in locations 1 and 4. The contrast of the image quality map of the IT specimen after creep testing in Figure 10a is reduced compared with the contrast before creep testing shown in Figure 3a. This change indicates that extensive recovery and growth of matrix grains took place after creep testing. The contrast is further reduced in the ITT specimen after creep testing, as shown in Figure 10d. The previous OTM grains (light) can barely be distinguished from the previous NTM grains (dark) after creep testing based on the brightness. Based on these image quality maps and inverse pole figure maps, the two specimens seem to be similar, with grain sizes of 2.63 µm and 2.55 µm for the IT and ITT specimens. However, the distributions of grain boundaries and KAM shown in Figure 10c,f indicate that the specimens are quite different. The fractions of the LAGBs are 52.9% in the IT specimen but only 35.0% in the ITT specimen. The KAM distribution in the ITT specimen is shifted to lower angles. The normalized KAM values are 0.90° in the IT specimen and 0.42° in the ITT specimen, respectively.

**Figure 11:**
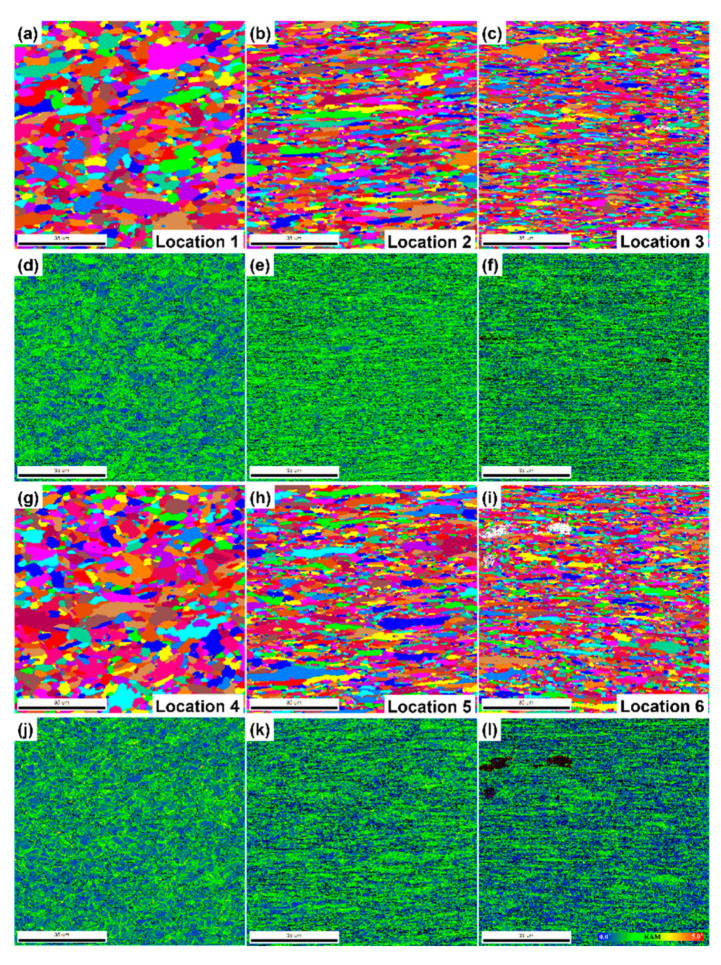
EBSD grain maps (**a**–**c**,**g**–**i**) and KAM maps (**d**–**f**,**j**–**l**) showing evolutions of grain size/morphology and local strain distribution in different locations 1–6 marked in Figure 7. All scale bars in figures are 35 μm.

**Figure 12:**
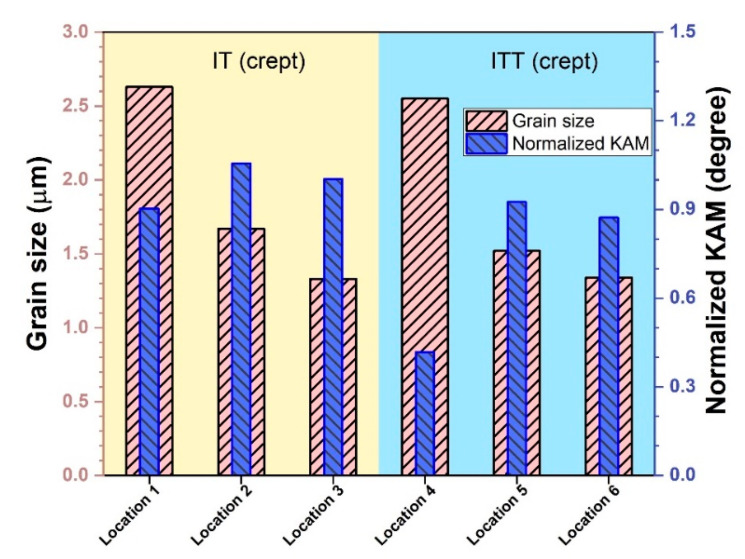
Comparison of grain size and normalized KAM values of different regions in the creep-ruptured specimens shown in Figure 11.

Overall, as shown in Figure 9, the precipitate density is higher in location 1 than in location 4, while the precipitate size is larger in location 4 than in location 1. The matrix grains in locations 2 and 5 are elongated horizontally along the creep location direction. Deformation of the matrix is slightly higher in location 5. Highly deformed fine grains, voids, and microcracks are observed in locations 3 and 6. Under severe creep deformation, the damage induced by inclusions is directly responsible for the ultimate failure [26]. The EBSD analysis illustrated in Figure 11 and Figure 12 reveals more features of these creep-ruptured specimens. Overall, the matrix grain size decreases between the undeformed locations 1 and 4 and the fractured locations 3 and 6: from 2.63 µm to 1.33 µm, respectively, in the IT specimen and from 2.55 µm to 1.34 µm, respectively, in the ITT specimen. This grain refinement was caused by recrystallization during fracture. Significant elongation/deformation of grains in locations 2 and 5 led to higher normalized KAM values (1.06° and 0.93°, respectively) than in the neighboring two locations.

## 4. Discussion

**Figure 13:**
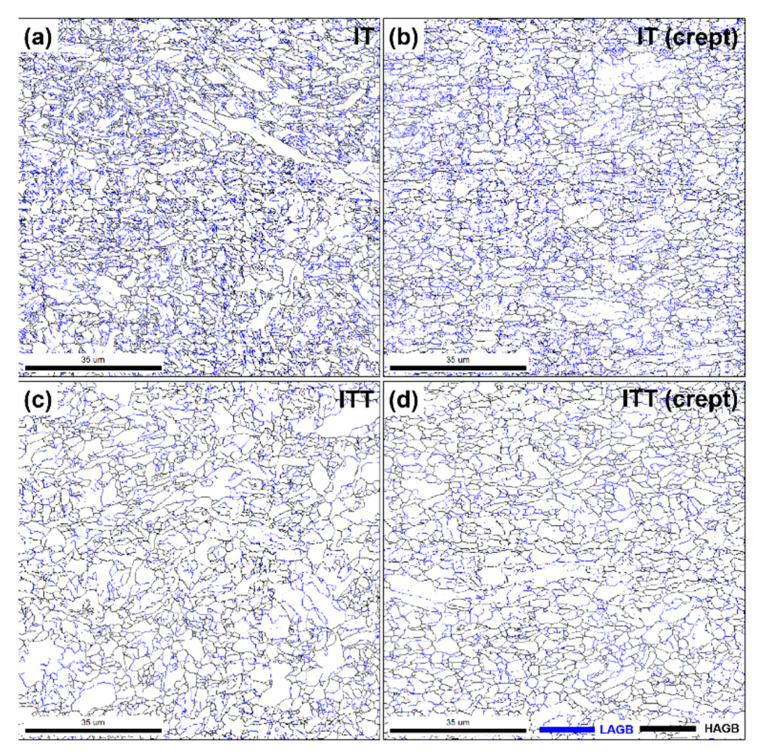
EBSD grain boundary maps of the IT and ITT specimens before (**a**,**c**) and after (**b**,**d**) creep testing.

The results presented in Section 3 clearly demonstrate that the evolutions of intercritical microstructures, consisting of untempered martensite and OTM, are dramatic under continuous thermal stages (PWHT and creep). The EBSD grain boundary maps in Figure 13 outline these evolutions well. Because of the limited peak temperature of the intercritical thermal cycle, the tempered martensite originating from the BM was only partially transformed into austenite. Quantification of the transformed martensite and untransformed martensite is challenging using conventional optical and SEM observations. The EBSD analysis approach presented in this work shows the success of visualizing and measuring the two kinds of microstructures, as shown in Figure 4. The untransformed tempered martensite was further tempered and is referred to as OTM. The OTM grains became ferritic grains with low dislocation densities, as shown by the low KAM values in Figure 3d. Figure 13a clearly shows that those blocky OTM grains (31.1%) are free of LAGBs, indicating that the multi-layer boundaries of martensite no longer exist. Figure 2b shows the OTM grains are almost free of precipitates as well. It is no surprise that these “free” OTM grains grew extensively during PWHT and creep testing owing to the missing pinning effects of precipitates and boundary barriers. Figure 13c shows the size of those blocky free grains greatly increases in the ITT specimen after PWHT. The average grain size continuously increases from 1.65 µm in the IT specimen to 1.99 µm in the ITT specimen and 2.55 µm in the creep-tested ITT specimen. Figure 13b shows that without PWHT, the free OTM grains greatly coarsen during short-term creep testing (32 h).

Two key features affect creep resistance of the partially transformed austenite grains and subsequent martensite. The intercritical peak temperature is not high enough for the transformed austenite grains to grow, resulting in a fine austenite (10 µm), as shown in Figure 2b. It has been reported that the numbers of transformed martensite packets and blocks decrease when the austenite grain size is below 10 µm [27,28]. It is obvious that creep resistance from grain boundary strengthening will be reduced with these reduced boundaries in martensite. It also has been observed that carbides as major carbon consumers were not fully dissolved inside those transformed austenite grains, as shown in Figure 2b. The incompletely dissolved carbides led to the formation of low-carbon austenite, which finally became martensite with a lower carbon content. During PWHT and creep, these transferred low-carbon martensite grains recovered and grew faster, owing to the lack of interstitial pinning by carbon. In Figure 10, obvious grain recovery and growth is observed. Another factor in this rapid evolution is the distribution of the undissolved precipitates. Figure 2b shows that they did not necessarily remain along the boundaries of the NTM. After PWHT, the precipitates coarsened but distributed inside the matrix instead of inside the grain boundaries, as shown in Figure 2d. Thus, these coarsened non-boundary precipitates did not provide sufficient precipitation strengthening.

Room temperature mechanical properties (hardness and tensile strength) are seen to be consistent with each other in Figure 5. The presence of NTM after the intercritical treatment is the dominating factor for increased hardness and tensile strength. The IT specimen shows the highest hardness and tensile strength among the three. A good balance between strength and ductility was achieved for the ITT specimen. Thus, this intercritical treatment might provide an alternative solution to improve room temperature toughness of martensitic steels. High-temperature creep resistance did not show the same trend as the room temperature properties. The BM with a moderate tensile strength showed the highest creep strength among the three specimens. The minimum creep strain rate of the ITT specimen (0.49 %/h) was 377 times the 0.0013 %/h rate of the BM and twice the 0.24 %/h rate of the IT specimen. The fast recovery and growth of low-carbon martensite and OTM grains led to low creep resistance in the IT and ITT specimens. In summary, the intercritical microstructures, including newly formed low-carbon martensite, OTM, and undissolved precipitates, were all found to be vulnerable to high-temperature creep deformation. The interactions between NTMs and OTMs under different creep temperatures and stress levels will be investigated in future studies. The microstructure evolutions promoted by high-temperature PWHT also accelerated creep strength degradation. Therefore, the selection of proper PWHT temperatures should be strictly based on calculated A_C1_ and A_C3_ temperatures of both the base metal and filler metal; otherwise, overheating may generate this intercritical microstructure and cause unexpected creep strength loss.

## 5. Conclusions

In this study, the microstructure and mechanical properties of intercritically treated Grade 91 steel were characterized and tested. The following points summarize the main findings.

1. Partial austenitization during the intercritical heat treatment led to the transformation of new martensite (69.9%) in the heat-treated specimen. The 31.1% of tempered martensite was over-tempered close to ferrite. Undissolved precipitates remained and coarsened in the NTM. The significantly increased hardness (332 HV0.5) and tensile strength (1054 MPa) were contributed by the hard NTM grains.
2. After additional tempering at 760 °C, a faster grain growth occurred in the OTM. The fine NTM laths recovered into fine equiaxed grains. Precipitate coarsening facilitated the grain growth due to the reduced pinning effect from coarser size and lower density, which led to a lowered hardness of 178 HV0.5 and a reduced tensile strength of 596 MPa.
3. The IT specimen showed slightly higher creep strength at 650 °C than the ITT specimen after additional tempering. However, both the IT and ITT specimens exhibited extremely low creep resistance compared with the BM. Severe creep deformation was observed on the creep-fractured specimens. Grain growth was still noticeable in the specimens that had undergone creep, even in this short-term creep test. Elongation of coarsened grains from the OTM contributed to plastic fracture of the specimens. Inclusions assisted the nucleation and growth of cavities and micro-cracks.

## Figures and Tables

**Table 1:** Chemical composition of the studied Grade 91 steel (wt. %).

**C**	**Mn**	**P**	**S**	**Si**	**Ni**	**Cr**	**Mo**
0.061	0.37	0.01	0.003	0.11	0.09	8.61	0.89
V	Nb	Ti	Co	Cu	Al	B	Fe
0.209	0.072	0.004	0.01	0.04	0.007	<0.09	Bal.

**Table 2:** Mechanical properties of the BM, IT, and ITT specimens.

Mechanical Properties	BM	IT	ITT
Microhardness (HV0.5)	247 ± 3	332 ± 12	178 ± 3
Yield strength (MPa)	611 ± 3	690 ± 1	419 ± 2
Ultimate tensile strength (MPa)	739 ± 1	1054 ± 8	596 ± 7
Elongation (%)	20	17	30
Creep fracture time (h)	170 (test terminated)	31.9	19.2
Minimum creep strain rate (%/h)	0.0013	0.24	0.49
